# User experiences and perceptions of health wearables: an exploratory study in Cambodia

**DOI:** 10.1186/s41256-021-00221-3

**Published:** 2021-09-23

**Authors:** Marco Liverani, Por Ir, Virginia Wiseman, Pablo Perel

**Affiliations:** 1 Department of Global Health and Development, London School of Hygiene and Tropical Medicine, London, UK; 2 School of Tropical Medicine and Global Health, Nagasaki University, Nagasaki, Japan; 3 Faculty of Public Health, Mahidol University, Bangkok, Thailand; 4 National Institute of Public Health, Phnom Penh, Cambodia; 5 The Kirby Institute, University of New South Wales, Sydney, Australia; 6 Centre for Global Chronic Conditions, London School of Hygiene and Tropical Medicine, London, UK

**Keywords:** Cambodia, NCDs, Health wearables, Fitness trackers, Health system strengthening

## Abstract

**Background:**

In many low- and middle-income countries (LMICs), health system capacities to address the burden of non-communicable diseases (NCDs) are often inadequate. In these countries, wearable health technologies such as smartbands and smartwatches could be used as part of public health programmes to improve the monitoring, prevention, and control of NCDs. Considering this potential, the purpose of this study was to explore user experiences and perceptions of a health wearable in Cambodia.

**Methods:**

Data collection involved a survey, conducted between November 2019 and January 2020, among different categories of participants (including hypertensive participants, non-hypertensive participants, postgraduate students, and civil servants). All participants were given a sample of a watch-type wearable and advised to use it day and night. One month after product delivery, we conducted a survey to explore their views and experiences. Results were analysed by using descriptive statistics and Chi square or Fisher's exact test to compare responses from urban and rural participants.

**Results:**

A total of 156 adult participants completed the study. Technology acceptance was positive overall. 89.1% of the participants said they would continue using the watch and 76.9% of them would recommend it to either friends or relatives, while 94% said the device stimulated them to think more frequently about their health. However, challenges to technology adoption were also identified, including concerns with the accuracy and quality of the device and unfamiliarity with the concept of health self-monitoring, especially among the elderly. Short battery life and cost were also identified as potential barriers to continued use.

**Conclusions:**

Health wearables are a promising new technology that could be used in Cambodia and in other LMICs to strengthen health sector responses to the challenges of NCDs. However, this technology should be carefully adapted to the local context and the needs of less resourced population groups. In addition, further studies should examine if adequate health sector support and infrastructure are in place to implement and sustain the technology.

**Supplementary Information:**

The online version contains supplementary material available at 10.1186/s41256-021-00221-3.

## Background

It is well known that low- and middle-income countries (LMICs) are experiencing a rising burden from non-communicable diseases (NCDs). In 2018, NCDs accounted for 41 million deaths worldwide and nearly 85% of premature deaths from NCDs occurred in LMICs [1]. Recent projections indicate that deaths due to NCDs in LMICs may exceed 70 million by 2060 [2]. In light of this, international and national efforts to address NCDs and known risk factors such as poor diet, tobacco use, and physical inactivity have increased. However, health system capacities to manage NCDs are often inadequate, with the financial burden of health expenditures falling on individuals and their families [3, 4]. Furthermore, health information systems in many LMICs are not designed to monitor the complexity of risk factors for NCDs at the individual and population level [5].

In such contexts, wearable health technologies such as smartbands and smartwatches could potentially be used as part of public health programmes to improve the awareness, prevention, monitoring, and control of NCDs. In recent years, a wide range of wearable devices have entered the consumer market, able to capture various biometric data, including heart rate, mobility, sleeping patterns, and calories spent. Furthermore, clinical grade devices with more advanced features such as blood pressure measurement, biomarkers for blood glucose, and hydration level are available [6]. Unlike data collection in clinical settings, these devices allow for continuous, unobtrusive, and ecologically valid data collection in real-world environments. As such, they could be deployed in both rural and urban communities to conduct regular surveys of risk factors for NCDs and their distribution across population groups, providing key evidence to inform policy development and programme implementation. In addition, health wearables could link users with the local health system, contributing to improved disease prevention, monitoring and management. However, successful adoption of health technologies is not only dependent on their fixed technical properties—it also requires sustained and appropriate use by motivated people [7]. Thus, an understanding of technology acceptance by target users in the communities is crucial to inform policy decisions and technology introduction. While studies of user experiences with health wearables have been conducted in high-income countries, little is known about the acceptance of this type of device in LMICs.

This paper addresses this research gap by presenting findings from a study of technology acceptance in Cambodia. Following decades of turmoil and civil conflicts, in the early 1990s the country embarked on a process of democratic transition, which laid the foundations for more representative governance. In the process, efforts were made to rebuild the health sector and to provide affordable care to all through pro-poor health insurance schemes [8]. Over time, these efforts have led to improvements in access to services and health outcomes, particularly in relation to infectious diseases, child and maternal health [9]. However, health sector development remains an important challenge and a development priority in Cambodia, particularly for the prevention and control of NCDs, which account for 64% of all deaths in Cambodia [1]. In line with global health policy, the Ministry of Health and international partners developed a national plan on NCDs amidst concerns that ﻿Cambodia will face “a tsunami of additional NCD patients in the coming years” [10]. Provisions for NCD management at the primary care level are however lagging [11, 12]. In addition, the health information system in Cambodia is not sufficiently developed to capture the breadth of data that are needed to monitor NCDs and associated risk factors [13]. At the same time, as in many other LMICs, access to internet and mobile technologies (including smartphones) has increased substantially in recent years [14], providing a fertile ground for the introduction of digital innovations and a relevant case study to inform policy and planning at the national and global level. Considering this potential, the study presented aimed to explore user experiences and perceptions of a health wearable in urban and rural Cambodia.

## Methods

### Conceptual framework

The study of the acceptance, adoption, and diffusion of technology has a long tradition in the social sciences [15–17]. Particularly relevant to this study were works and theories that focus on the end users in processes of technology adoption, such as the Technology Acceptance Model (TAM) [18]. The TAM was developed in the late 1980s to improve the design of early IT solutions in the business sector, drawing on theories in social psychology about the link between attitude and human behaviour [19]. In its original formulation, this model defined technology acceptance as the intention of individuals to use that technology, which in turn is influenced by two key variables: perceived usefulness and perceived ease of use. Perceived usefulness was defined as “the extent to which a person believes that using the technology would enhance her or his performance”, while perceived ease of use was “the extent to which a person believes that using the technology will be free of effort or hurdles” [18]. Since then, this approach has been refined and applied widely to assess a variety of technologies in the private and public sectors [20]. In studies of mHealth, the original emphasis on “performance” has shifted towards improved health, wellbeing, or access to care and health services [21]. In addition, specific attention is paid to health-related variables that may influence users’ perceptions such as health status, health-seeking behaviour, and previous exposure to digital health applications [22–24].

Our study was also guided by sociological perspectives on technology adoption, which emphasise the need to move beyond simple dichotomies such as use and non-use [25]. Rather, a range of utilisation patterns and users’ preferences can often be observed among users of the same technology, especially when multiple functions and applications are embedded in technology design as is the case of health wearables [26]. Furthermore, it is well documented that gaps in education, income, and access to resources may be significant barriers to technology adoption in LMICs [27]. In Cambodia these concerns are crucial given wide disparities in wealth and persisting poverty, against a backdrop of rapid economic growth [28]. Similarly, differences in socio-economic status and income may affect the ability of users to pay for health services and technologies, with negative effects on programme implementation [29]. Thus, we also considered the willingness to pay (WTP) for the proposed technology, defined as the “maximum sum an individual (or a government) is willing to pay to acquire a good or service, or the maximum sum an individual (or a government) is willing to pay to avoid a prospective loss” [30].

### Research design and participants

Drawing on the concepts and considerations outlined above, we designed and conducted a survey to explore users’ perceptions and experiences with a watch-type health wearable in Cambodia, including: (1) perceived usefulness and ease of use; (2) utilisation patterns; (3) intention to use; and (4) willingness to pay. The sample wearable was produced by a manufacturer in Shenzhen, China, and could be used to tell the time and date, measure heart rate, blood pressure, steps, and track calories through an entirely graphical interface. The device could be paired to a dedicated smart phone application providing basic statistics of user data and trends over time.

In order to account for potential rural/urban differences, the survey was conducted in Phnom Penh, Cambodia’s capital, and Kampot, a rural province in the southwest of the country. In both locations, all participants were provided with a sample device and advised to wear it as much as possible, day and night. One month after product delivery, the same participants were interviewed to examine their views and experiences with the device. Given the exploratory nature of our study and limited supply of the study watch, we did not use representative sampling but we aimed to capture diversity through purposive quota sampling focusing on the following categories: (1) adult participants with diagnosed hypertension in Kampot; (2) adult participants without diagnosed hypertension in Kampot; (3) adult participants with diagnosed hypertension in Phnom Penh; (4) adult participants without diagnosed hypertension in Phnom Penh. The sample frame of households for the random selection of participants was obtained from a local non-governmental organization (NGO) providing free health consultations and support in local communities. In Phnom Penh, we also recruited postgraduate university students and civil servants working within the Ministry of Health to test if higher educational background and health literacy could be associated with differences in users’ experiences.

#### Questionnaire development

The survey consisted of two custom questionnaires (Additional file 1), aligned with our research objectives, the conceptual framework outlined above, and the technical specifications of the sample device. The first questionnaire, administered at product delivery, included questions on self-reported health status, diet, and health-seeking behaviour, adapted from standard health surveys [31, 32] after discussion within the research team. At the end of the questionnaire, information on socio-economic status was collected to construct socio-economic tertiles, using the EquityTool for Cambodia [33]. The EquityTool is a short, country-specific questionnaire, developed and validated to assess relative wealth based on an asset index [34].

One month after product delivery, a second questionnaire was administered to the same sample of participants. The second questionnaire aimed to assess technology acceptance and how participants had used the device, their views about its most useful features, practical usability issues including potential discomfort while wearing the watch or any other barriers to continued use, and willingness to pay (WTP) for the watch. The questions about product acceptance were adapted from those developed in TAM studies [18, 35] and previous research on wearables [36], considering the technical specification of the sample device and the research context. Given the exploratory nature of the survey, open-ended questions were also included to encourage spontaneous input from the participants. Lastly, estimates of WTP were elicited using an iterative “bidding game” approach, a method widely used to study the economic value of non-market goods in health care [37–39]. The two questionnaires were developed in English and subsequently translated into Cambodian to enable collaboration within the research team. The draft questionnaires were piloted and back translated to improve internal consistency and refine the formulation of questions.

#### Data collection and processing

The finalised surveys were conducted between November 2019 and January 2020 by four Cambodian researchers, with training in health research methods. The wearables were provided free of charge to all participants. At product delivery, the researchers provided participants with oral and written instructions on how to use the watch and the paired application. Data collected on paper forms were entered into an Excel file, and then imported into STATA version 13.1 for cleaning, processing, and statistical analysis.

#### Statistical analysis

The statistical analysis was performed using Stata version 16 (Stata Corp, College Station, TX, USA). Descriptive statistics were used to summarize respondent characteristics and outcomes in product utilisation and acceptance in the follow-up survey. Chi square or Fisher's exact test (p value less than 0.05) were used to compare the demographic and socio-economic profiles of urban and rural participants. Textual information from open-ended responses was coded for frequency analysis and translated from Cambodian into English. The Kruskal–Wallis test was used to test for significant differences in mean WTP across socioeconomic groups.

## Results

### Demographic and socio-economic characteristics

A total of 156 adult participants completed the study. These included 60 hypertensive participants (30 in Phnom Penh and 30 in Kampot), 60 non-hypertensive adults (30 in Phnom Penh and 30 in Kampot), 18 civil servants and 18 postgraduate students in Phnom Penh. Only one participant at baseline (a civil servant in Phnom Penh) did not complete the follow-up survey due to watch malfunction.

Table 1 shows the demographic and socio-economic characteristics of the participants. On average, participants were aged 53.2 years (SD: 14.5; range: 22–79). In Phnom Penh, 59.8% of participants had some secondary or higher education and 52.6% were in the highest socio-economic status group. By contrast, most participants in Kampot had attended only primary school (48.3%) and were in the lowest socioeconomic group (71.7%).

**Table 1:** Demographic and socio-economic characteristics of participants

	Phnom Penh n (%)	Kampot n (%)	Totals n (%)	*p* value
Age group (years)^a^				0.000
< 30	23 (23.7)	–	23 (14.7)	
31–40	11 (11.3)	1 (1.7)	12 (7.7)	
41–50	4 (4.1)	7 (11.7)	11 (7.0)	
51–60	31 (32.0)	24 (40.0)	55 (35.0)	
61–70	25 (25.8)	21 (35.0)	46 (29.3)	
> 70	3 (3.1)	7 (11.7)	10 (6.4)	
Sex^b^				0.851
Female	50 (51.6)	30 (50.0)	77 (49.0)	
Male	47 (48.4)	30 (50.0)	80 (51.0)	
Education^b^				0.023
No education	13 (13.4)	6 (10.0)	19 (12.1)	
(Some) primary	26 (26.8)	29 (48.3)	55 (35.0)	
(Some) secondary and higher	58 (59.8)	25 (41.7)	83 (52.8)	
HH socio-economic status^a^				0.028
Worse off	10 (10.3)	43 (71.7)	53 (33.7)	
Middle	36 (37.1)	17 (28.3)	53 (33.7)	
Better off	51 (52.6)	–	51 (32.5)	
Own a smartphone^b^	75 (77.3)	27 (45.0)	102 (65.0)	0.000

^a^Fisher’s exact test

^b^Chi square

These differences in socio-economic status were mirrored in the ownership of assets, including smartphones. While 77.3% of respondents in Phnom Penh owned a smartphone, only 45% in Kampot did. Of those who did not own a smartphone, the large majority (94.6%) were over 50 years of age.

### Health and wellbeing

Table 2 summarises findings on self-reported health status, health behaviour and self-monitoring of health and fitness. At product delivery, 3.9, 30.6, 60.5 and 5.1% of the participants rated their health as “very good”, “good”, “fair” and “poor” respectively. Of those who rated their health as “fair” or “poor”, the majority were hypertensive (46.3%) and above 50 years of age.

**Table 2:** Self-reported health and health seeking behaviour

	Phnom Penh n (%)	Kampot n (%)	Totals n (%)	*p* value
Do you smoke?^a^				0.828
No	84 (86.7)	50 (83.3)	134 (85.4)	
Occasionally	5 (5.2)	3 (5.0)	8 (5.1)	
Daily	8 (8.3)	7 (11.7)	15 (9.6)	
How would you rate your health overall?^a^				0.007
Very good	6 (6.2)	–	6 (3.8)	
Good	36 (37.1)	12 (20)	48 (30.6)	
Fair	52 (53.6)	43 (71.7)	95 (60.5)	
Poor	3 (3.1)	6 (8.3)	8 (5.1)	
How often do you do physical exercise?^b^				0.433
Never	14 (14.4)	13 (21.7)	27 (17.2)	
Occasionally	36 (37.1)	18 (30.0)	54 (34.4)	
Every week, at least once	47 (48.5)	29 (48.3)	76 (48.4)	
How often do you do checks with health care providers?^a^				0.007
Never	21 (21.7)	3 (5.0)	24 (15.3)	
Once a year	22 (22.7)	11 (18.3)	33 (21.0)	
More than once a year	53 (54.6)	46 (76.7)	99 (63.0)	
Don’t know	1 (1.0)	–	1 (0.6)	
How often do you check your blood pressure at home?^a^				0.000
Never	47 (48.5)	50 (83.3)	97 (61.8)	
Rarely	9 (9.3)	–	9 (5.8)	
Regularly	38 (39.2)	10 (16.7)	48 (30.6)	
Don’t know	3 (3.1)	–	3 (1.9)	
Ever used a fitness watch	11 (11.3)	–	11 (7.0)	
Ever used a mobile fitness/health app	5 (5.2)	–	5 (3.2)	

^a^Fisher’s exact test

^b^Chi square

With respect to the behavioural questions, 85.4% of participants reported to be non-smokers, 51% said they would eat fruit or vegetables at least once a day, and 48.4% did physical exercise “every week, at least once”. When asked about their preventive care behaviour, 90% of hypertensive participants said they attended check-ups with a doctor, nurse or other health care provider more than once a year. However, only 46.4% of non-hypertensive participants reported regular visits and 16.2% of those older than 50 said they would never do preventive health checks. Lastly, only 3.2% of participants reported previous use of a smartphone fitness application and only 7% had used a health wearable before the survey—all of them in Phnom Penh and in their 20s (81.8%) or 30s (18.2%). Most hypertensive patients (61.7%) said they had never self-monitored their blood pressure at home and none of them had ever used a mobile health application or fitness watch.

### Product utilisation, perceived usability and usefulness

Although health wearables were new to most participants, the technology acceptance was positive overall. 89.1% said they would continue using the watch and 76.9% would recommend it to either friends or relatives, while 94% said the device stimulated them to think more frequently about their health (Table 3).

**Table 3:** Utilisation and perceived usefulness of the study wearable

	Phnom Penh n (%)	Kampot n (%)	Totals n (%)	*p* value
How often have you used the watch in the past month?^a^				0.571
Sometimes	63 (66.0)	42 (70.0)	105 (67.3)	
Most of the time	33 (34.0)	18 (30.0)	51 (32.7)	
Do you feel this watch made you think about your health more than usual?^b^				1.000
Yes	89 (92.7)	57 (95)	146 (94.0)	
No	4 (4.2)	2 (3.3)	6 (3.9)	
Do not know	3 (3.1)	1 (1.7)	4 (2.6)	
Would you continue using this watch?^b^				0.111
Yes	82 (85.4)	57 (95.0)	139 (89.1)	
No	10 (10.4)	3 (5.0)	13 (8.3)	
Do not know	4 (4.2)	–	4 (2.6)	
Would you recommend this product?^a^				0.886
Yes	67 (77.9)	43 (75.4)	110 (76.9)	
No	12 (14.0)	8 (14.0)	20 (14.0)	
Do not know	7 (8.1)	6 (10.5)	13 (9.0)	

^a^Chi square

^b^Fisher’s exact test

Despite this positive attitude towards the device, only 34% of participants used the watch all the time during the study period, as instructed. When asked to provide further explanation, the most frequently mentioned barrier to utilisation was low battery life (n = 75/186), which lasted 3.9 days on average (SD = 2.9). Other reasons that were frequently given to explain sporadic use included watch malfunction (n = 28/186), the inconvenience of wearing the watch while taking a bath or shower (n = 36/186) and farming (n = 19/186). A minority of participants (n = 3) also complained they were “annoyed” by the watch, while others said the display was too small. Of note, two participants reported “pain in the arm” and a “sense of tingling in the chest” after wearing the watch.

**Fig. 1:**
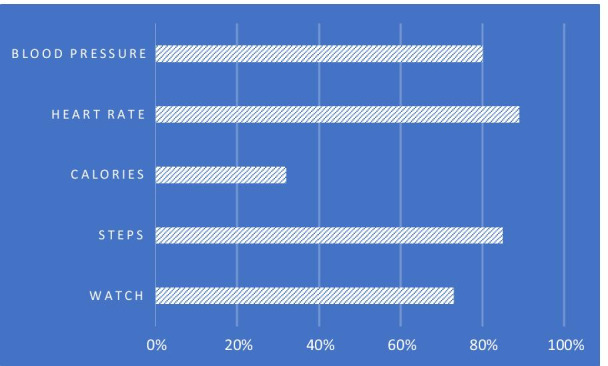
Self-reported utilisation of the wearable by different functions

Specific features of the wearable device such as the measurement of heart rate, steps, and blood pressure were used by 89.1, 85,3 and 80.1% of participants respectively. Only 32,1% used the calories tracker (Fig. 1). In keeping with this pattern, when asked to name the most useful functions, the majority of participants (62.8%) named blood pressure measurement as the first choice, followed by heart rate monitor as the second choice (48.1%) and step counter as the third choice (19.9%). As expected, being hypertensive or older than 45 years was significantly associated with a preference for blood pressure measurement. Only a minority of participants (25.6%) used the linked smartphone application, largely students (84.2%) and civil servants (72.2%).

Lastly, when asked if they had any suggestions for improvement, most participant mentioned that the watch should be more resistant to water or scratches (n = 56/123) and its battery life (n = 49/123) could be improved.

### Willingness to pay

Considering the potential marketing of a similar product as part of a public health programme, we were interested in exploring how much people would be willing to pay for it. In both study locations, respondents were willing to purchase the device for an average price of 46,774 Cambodian riels (US$ 11.4 Range: US$ 2.4–48.7). However, only 60% of participants were willing to buy the product. Socioeconomic status had a statistically significant effect on mean scores of WTP (Table 4).

**Table 4:** Willingness to pay for the wearable (n = 93/156) by socio-economic status, Cambodian riel (US Dollar)

	Mean	SD	Median	Range	*P* value
Willingness to pay					0.0013 ^*^
1 (Worse off)	35,417 (8.6)	14,161 (3.5)	40,000 (9.8)	10,000 (2.4)–70,000 (17.1)	
2 (Middle)	42,917 (10.5)	18,053 (4.4)	40,000 (9.8)	10,000 (2.4)–80,000 (19.5)	
3 (Better off)	61,970 (15.1)	42,388 (10.3)	50,000 (12.2)	20,000 (4.9)–200,000 (48.7)	
Total	46,774 (11.4)	30,330 (7.4)	40,000 (9.8)	10,000–(2.4) 200,000 (48.7)	

^*^Kruskal–Wallis test (Χ^**2**^ = 13.304)

## Discussion

This paper examined users’ experiences with wearable health trackers in Cambodia, contributing new insights into the acceptance of this technology and the wider study of mHealth in LMICs. As described, most participants had little or no experience with wearable health trackers or smartphone applications for health monitoring prior to the study, with the exception of some postgraduate students in Phnom Penh. Nonetheless, the large majority of participants reported a positive experience with the device, increased health awareness and a willingness to use and recommend the device to other people after the study period. In general, participants found the health wearable useful, suggesting a similar device would be well received and could be used as a tool to monitor and control risk factors for NCDs in Cambodia, including in rural areas where access to preventive care for NCDs is more limited [40].

Our study also indicates that product design and features should be tweaked to maximise technology acceptance and utilisation. As we have seen, the need to charge the device was off-putting for many participants, suggesting that a self-charging battery would likely increase utilisation, particularly amongst households with limited access to electric power. Furthermore, our study found that only 45% of participants in Kampot owned a smartphone. This is consistent with a previous survey of mobile phone use in Cambodia, which found that smartphone ownership increased but was still relatively low in rural areas (42%) [14]. Thus, a standalone device that can be fully operated without smartphone support would be more suitable for wide use. Lastly, a solid, waterproof design would appeal to those participants, particularly farmers, that were concerned about water damage.

Further consideration of the study findings and the study context highlights other potential challenges to technology adoption. In particular, the use of consumer health wearables is premised on an individualistic concept of care in which “digitally engaged patients” are expected to manage their own preventive health efforts [41]. Even if wearables can be designed to deliver messages and reminders based on the analysis of user data, continued use still requires a commitment to actively incorporate self-care into daily routines. In Cambodia, this may conflict with traditional culture and social norms, which emphasise the collective, social dimension of caring and disease management, particularly for the elderly. In this respect, it is worth noting that most hypertensive participants in our sample reported having regular check-ups with health providers but only a few were used to monitoring their own blood pressure.

Cost may be another important barrier to product adoption amongst poorer populations. While the average willingness to pay was high (US$ 11.4) relative to an annual household income per capita of US$ 1,530 (World Bank 2019), many participants in the lowest tertile were willing to pay only a fraction of the estimated market value (which is about US$ 30) and less than two thirds were willing to buy the watch. Thus, wide technology adoption would require some form of subsidisation or the development of a lower-cost technology, bearing in mind that participants in our study were sensitive to product design and quality. Alternatively, a public–private partnership could be devised to reduce costs and increase participation, as seen recently in Singapore [42]. In 2019, Fitbit, a leading manufacturer of consumer wearables, partnered with the government of Singapore to develop a large public health program seeking to better understand the health behaviours and lifestyles of Singapore residents using wearable technologies. Under this program, participants are given a Fitbit smartband for free, provided they consent to sharing their data with Singapore’s Health Promotion Board, a government agency under the Ministry of Health which uses collected information to carry out large studies of population health and health risks [42]. In Cambodia, a similar arrangement could be made, although adequate regulations and technical safeguards should be in place to ensure the protection of data privacy.

Lastly, any new technology is just one component of sustainable development along with other important domains such as human resources and wider infrastructure. In recent years, for example, the One Laptop per Child initiative distributed low‐cost “children machines” designed to empower youth in LMICs to learn without their schools and teachers [43]. The rationale was that ﻿efforts to reform curricula in some low-income countries were too slow or expensive and teacher training was seen as of limited value due to teacher absenteeism. This program was partly successful, but only in contexts where other key gaps were addressed, including increasing school attendance by teachers and students and dissemination of course materials [44]. Similarly, health wearables alone are unlikely to have any significant impact on health outcomes in Cambodia and elsewhere. Sustainable program implementation would also require health system integration, public funding, and improvements in the quality of care, which remains a significant challenge in Cambodia [45]. In recent years, other mHealth interventions have been piloted in Cambodia including smartphones applications to deliver messages for hypertensive and diabetic patients [46], to improve newborn care awareness in rural areas [47], to remind users about available family planning methods [48], and to support community-based malaria surveillance [49]. While these programmes have generally had a positive impact on health outcomes, sustainability of donor-driven initiatives has been a recurrent challenge. By contrast, local production of technology and the involvement of local entrepreneurs are more likely to increase local relevance, access, and ownership, while preventing undesirable outcomes such as the prioritisation of commercial interests of foreign companies over local public health needs [50].

This study has some limitations. The small sample size is a clear limitation of this study. In addition, the survey questionnaires were largely structured, with only a few open-ended questions. Therefore, we could not gain in-depth qualitative insights into individual perceptions and experiences with the given technology. The use of qualitative methods would be particularly useful to explore in greater depth different patterns of technology acceptance between urban and rural participants. This exploratory study was also carried out over a relatively short period due to time and resource constraints. As a result, we could not examine phenomena that would require a longer timeframe such as behaviour change and potential changes over time in user perceptions about the technology. For the same reason, we could not extend our analysis from the *adoption intention* to *technology appropriation*, described as “the process whereby one or more users makes a technological artifact or system theirs, integrating it into their sociocultural world and in the process transforming said artifact or system to serve the user’s ends” [51]. This would be particularly important also considering previous studies that found a gradual decline in the use of these devices or even abandonment within a few months after purchase [52]. Finally, the study methodology relied on self-assessed measures of health status and determinants, which are prone to recall and other subjective biases [53].

## Conclusions

This study provides new insights into the acceptance of health wearables in a low-resource context and its potential to support public health efforts to reduce the burden of NCDs. The research findings suggest that the introduction of health wearables as part of a public health programme in Cambodia could contribute to strengthening the monitoring and control of NCDs and associated risk factors, although product design, features, and costs should be adapted to the local context. As discussed, a self-charging device, water and shock resistant, which can be fully operated without smartphone support, would be appealing to a larger share of the population, especially in rural areas. Further evaluations should be conducted to provide a robust assessment of impact, comparing for example key outcomes between users and a control group of non-users, to see if there are different outcomes in terms of risk behaviour and health seeking behaviour. Studies of health system and policy variables that may influence technology adoption would also be needed to inform programme development and implementation (Additional file 1).

## Supplementary Information

**Additional file 1.** Survey questionnaires.

## Data Availability

Please contact corresponding author for data requests.

## Abbreviations

LMICs: Low- and middle-income countries
NCDs: Non-communicable diseases
NGO: Non-governmental organization
TAM: Technology acceptance model
WTP: Willingness to pay
