# Supplementary material for: User experiences and perceptions of health wearables: an exploratory study in Cambodia
Source: Glob Health Res Policy. 2021 Sep 23;6:33. doi: 10.1186/s41256-021-00221-3 (PMC8459510; doi:10.1186/s41256-021-00221-3)
Supplement: Supplementary file 1 — Additional file 1. Survey questionnaires. [file 41256_2021_221_MOESM1_ESM.docx]

**Baseline survey**

| **Demographic information** | | |
| --- | --- | --- |
| **Code** | **Question** | **Response** |
| C1 | Sex (as observed) | \| Male \| 1 \| \| --- \| --- \| \| Female \| 2 \| |
| C2 | How old are you? | Years __ __ |
| C3 | In total, how many years have you spent at school (excluding pre-school)? | Years __ __ |
| C4 | Which of the following best describes your **main work** status over the past 12 months?  (USE SHOWCARD) | 1. Employed (public sector) 2. Self-employed (small business) 3. Farmer 4. Employed (private sector) 5. Unemployed/causal worker 6. Unpaid family/household worker 7. Retired 8. Student 9. Other (Specify ________)   97. Don’t know |

| **Socio-economic status** | | |
| --- | --- | --- |
| **Code** | **Question** | **Response** |
| Q1 | Does your household have electricity? | 1. Yes 2. No |
| Q2 | …a television? | 1. Yes 2. No |
| Q3 | …a refrigerator? | 1. Yes 2. No |
| Q4 | …CD/DVD player? | 1. Yes 2. No |
| Q5 | …wardrobe? | 1. Yes 2. No |
| Q6 | …generator/battery/solar panel? | 1. Yes 2. No |
| Q7 | Does any member of your household own a… motorcycle/scooter? | 1. Yes 2. No |
| Q8 | …a watch? | 1. Yes 2. No |
| Q9 | Do you or any other member in your household have a bank account? | 1. Yes 2. No |
| Q10 | What is the main source of drinking water during the wet season for members of your household? | 1. Piped into dwelling 2. Other water source |
| Q11 | What is the main material of the floor? | 1. Ceramic tiles 2. Wood planks 3. Other material |
| Q12 | What is the main material of the exterior walls? | 1. Cement blocks 2. Palm/bamboo/thatch 3. Other material |
| Q13 | What type of fuel does your household mainly use for cooking? | 1. LPG 2. Wood 3. Other type of fuel |
| Q14 | What kind of toilet facility do you usually use? | 1. Flush to piped sewer system (not shared with other households) 2. Flush to septic tank (not shared with other households) 3. No facility / bush / field 4. Other type of toilet |

| R1 | Do you have a mobile phone? | 1. Yes 2. No -> **P1** |
| --- | --- | --- |
| R2 | Is it a smartphone? | 1. Yes 2. No |

| **Health and wellbeing** | | |
| --- | --- | --- |
| **Code** | **Question** | **Response** |
| P1 | Do you smoke? | 1. No 2. Occasionally 3. Daily |
| P2 | How often do you eat fruit or vegetables? | 1. Not very often 2. Every week 3. Every day, at least once 4. More than once a day 5. Don’t know |
| P3 | How often do you do physical exercise (such as swimming, cycling, running, fitness, other recreational activities such as volleyball or football)? | 1. Never 2. Occasionally 3. Every week, at least once |
| P4 | On average, how many hours do you sleep at night? | Hrs _____ |
| P5 | Do you do routine health checks with a doctor, nurse or other healthcare provider? | 1. Never 2. Rarely 3. Regularly 4. Don’t know |
| P6 | Do you do any self-monitoring of your health status (such as blood pressure measurement)? | 1. Never 2. Rarely 3. Regularly 4. Don’t know |
| P7 | Have you ever used a fitness watch or a mobile health app? | 1. Yes 2. No -> P9 |
| P8 | Did (or do) you find it useful? Can you explain? |  |
| P9 | In general, how would rate your health overall? | 1. Excellent 2. Very good 3. Good 4. Fair 5. Poor |

**Follow-up Survey**

| **Product understanding and reception** | | |
| --- | --- | --- |
| **Question** | **Response** | **Code** |
| How often have you used the watch in the past month? | 1. Never -> A1S 2. All the time 3. Sometimes 4. Don’t know | A1 |
| Why didn’t you wear the watch at all? Can you explain? | -> END OF INTERVIEW | A1S |
| Did you use the watch to check any of the following?  (USE SHOWCARD) | 1. Time 2. Steps 3. Calories 4. Heart rate 5. Blood pressure 6. None ->A9 | A2 |
| Can you list the three most useful applications (in order)? | 1. ________________  2. ________________  3. ________________ | A3 |
| Did you think about your health more than usual in the past month? | 1. Yes 2. No 3. Don’t know | A4 |
| Did you have any discomfort while wearing the watch? | 1. Yes 2. No -> A6 | A5 |
| Can you explain what kind of discomfort you had? |  | A5a |
| Do you have any suggestions on how the watch could be improved? | 1. Yes 2. No -> A7 | A6 |
| Can you explain how it could be improved? |  | A6a |
| Did you use the associated app on your mobile phone? | 1. Yes 2. No -> A9 | A7 |
| Was the app easy to use? How would you rate it? | 1. Very easy 2. Easy 3. Difficult 4. Very difficult 5. Don’t know | A8 |
| How long did the battery last for? | Days ___ ___ | A9 |

| **Willingness to pay** | | |
| --- | --- | --- |
| **Question** | **Response** | **Code** |
| Would you continue to use this watch? | 1. Yes  2. No **→ END OF INTERVIEW**  3. Don’t know | C1 |
| Would you be willing to buy this watch? | 1. Yes  2. No **→ END OF INTERVIEW**  3. Don’t know | C2 |
| Would you be willing to pay US$ 10 for this watch? | 1. Yes  2. No **→ C5**  3. Don’t know | C3 |
| Would you be willing to pay US$ 30 for this watch? | 1. Yes **→ C6**  2. No **→ C6**  3. Don’t know **→ C6** | C4 |
| Would you be willing to pay US$ 5 for this watch? | 1. Yes  2. No  3. Don’t know | C5 |
| What is the highest price you would be willing to pay for this watch? | 1. Riel ___________  2. Don’t know | C6 |
| Would you recommend others to use this watch? | 1. Yes  2. No **→ END OF INTERVIEW**  3. Don’t know **→ END OF INTERVIEW** | C7 |
| Who is the person you would recommend this product? |  | C8 |
